# *PLAGL1* gene function during hepatoma cells proliferation

**DOI:** 10.18632/oncotarget.25996

**Published:** 2018-08-28

**Authors:** Ana F. Vega-Benedetti, Cinthia N. Saucedo, Patrizia Zavattari, Roberta Vanni, Felix Royo, Francisco Llavero, José L. Zugaza, Luis A. Parada

**Affiliations:** ^1^ Institute of Experimental Pathology, CONICET-UNSa, Salta, Argentina; ^2^ Biochemistry, Biology and Genetics Unit, Department of Biomedical Sciences, University of Cagliari, Cittadella Universitaria di Monserrato SP 8, Monserrato, Cagliari, Italy; ^3^ CIC BioGUNE-CIBERehd, Bizkaia Technology Park, Derio, Spain; ^4^ Achucarro Basque Center for Neuroscience, UPV/EHU Technology Park, Leioa, Spain; ^5^ Department of Genetics, Physical Anthropology and Animal Physiology, Faculty of Medicine and Dentistry, University of the Basque Country, Leioa, Spain; ^6^ IKERBASQUE, Basque Foundation for Science, Bilbao, Spain

**Keywords:** PLAGL1, hepatocellular carcinoma, cell proliferation, chromosome, methylation

## Abstract

Hepatocellular carcinoma develops as a multistep process, in which cell cycle deregulation is a central feature, resulting in unscheduled proliferation. The *PLAGL1* gene encodes a homonym zinc finger protein that is involved in cell-proliferation control. We determined the genomic profile and the transcription and expression level of *PLAGL1*, simultaneously with that of its molecular partners *p53*, *PPARγ* and *p21*, in cell-lines derived from patients with liver cancer, during *in vitro* cell growth. Our investigations revealed that genomic and epigenetic changes of *PLAGL1* are also present in hepatoma cell-lines. Transcription of *PLAGL1* in tumor cells is significantly lower than in normal fibroblasts, but no significant differences in terms of protein expression were detected between these two cell-types, indicating that there is not a direct relationship between the gene transcriptional activity and protein expression. RT-PCR analyses on normal fibroblasts, used as control, also showed that *PLAGL1* and *p53* genes transcription occurs as an apparent orchestrated process during normal cells proliferation, which gets disturbed in cancer cells. Furthermore, abnormal trafficking of the PLAGL1 protein may occur in hepatocarcinogenesis.

## INTRODUCTION

Hepatocellular carcinoma (HCC) is the sixth most common cancer and the third cause of death cancer worldwide. HCC is widely distributed around the world, presenting higher incidence in sub-Saharan Africa and Eastern Asia than Northern Europe, Oceania and America [1]. This uneven geographical distribution is closely related to that of the risk factors which vary among regions. For instance, the hepatitis B and C virus infections are common in Asia and Japan, respectively, whereas obesity and non-alcoholic liver disease lead to an increased incidence of HCC in United States and other western countries [2].

HCC develops as a multistep process, in which multiple factors play a role creating the conditions for malignant transformation. For example, hepatitis B or C viruses infection lead to liver damage and regeneration events, in which the persistent inflammation and oxidative stress favour the occurrence of genomic instability leading to the accumulation of genetic and epigenetic alterations that are a hallmark of tumorigenesis [3, 4]. Cell proliferation is regulated by a complex network of signalling pathways, such as HGF/MET, Wnt/β-catenin and p53, among others [5, 6]. Structural and/or functional alterations of proteins that participate in these pathways, leading to cell-cycle deregulation are frequently found in HCC cells [7]. For example, mutations in the *CTNNB1* gene, encoding for an abnormal β-Catenin protein, have been found in about 30% of HCC biopsies analyzed by Schulze *et al.* in 2016 [8]. While *in vitro* studies, using the HepG2, SkHep1 and Huh7 cell lines derived from human heptomas demonstrated that down-regulation of the *COMMD7* gene and the treatment with isocorydine and interferons favour inhibition of cell proliferation and induction of apoptosis [9–11].

The *PLAGL1* (Pleiomorphic Adenoma Gene-Like 1) gene maps on chromosome 6q24 [12], and it encodes a homonym zinc finger protein that functions as a transcription factor and as a cofactor of other proteins involved in cell cycle control [13]. PLAGL1 carries on its activities through convergent mechanisms. On one hand, it interacts with p53 and this heterodimer induces the expression of the receptor for pituitary adenylyl cyclase-activating peptide (PACAP_1_-R). The binding peptides to PACAP_1_-R induce gene transcription through AP-1, essential for proliferation and differentiation of various cell types [14]. Moreover, PLAGL1 and p53 bind as a complex to the promoter of *p21* gene, an important cell cycle regulator; favouring its transcription and leading to cell cycle arrest in G1 phase [15, 16]. On the other hand, PLAGL1 induces the expression of PPARγ that inhibits cell cycle progression through p21 induction and metastatic activity through the regulation of matrix metalloproteinases expression [17, 18]. It was demonstrated that genomic changes such as loss of heterozygosity (LOH) and hypermethylation of the P1 promoter of the *PLAGL1* gene are frequently observed in several types of cancer such as pheochromocytoma [19], ovarian cancer [20], breast cancer [21], pituitary adenomas [22] and hemangioblastoma [23], and in tumor cell lines including breast cancer cell lines [21]. Moreover, altered expression of *PLAGL1*, mainly reduced, was revealed in colorectal cancer and non-functioning pituitary adenoma [24, 25]. Midorikawa *et al.* examined samples of HCC, and found that LOH at chromosome 6q, hypermethylation of *PLAGL1* promoter at the remaining allele and low RNA expression levels were present in their series [26]. Since it was first described, the gene has been considered a tumor suppressor gene (TSG) [27], and all this evidence provided support for such classification. However, overexpression of *PLAGL1* was detected in some human neoplasms such as glioma and clear cell renal cell carcinoma suggesting an oncogenic function, as well [28, 29].

In the present study we investigated the profile of 6q2 aberrations, where *PLAGL1* gene maps, in four hepatoma cell-lines and the transcription and protein expression level of *PLAGL1* and its molecular partners *p53*, *PPARγ* and *p21* during *in vitro* cell-proliferation. Our data confirm that genomic and epigenetic changes of *PLAGL1* are also present in HCC cell-lines. Furthermore, we found that there is not a direct relationship between the gene transcriptional activity and protein expression during cell-proliferation and that abnormal subcellular localization of the PLAGL1 protein may occur during hepatocarcinogenesis.

## RESULTS

### Array-CGH analysis

**Figure 1:**
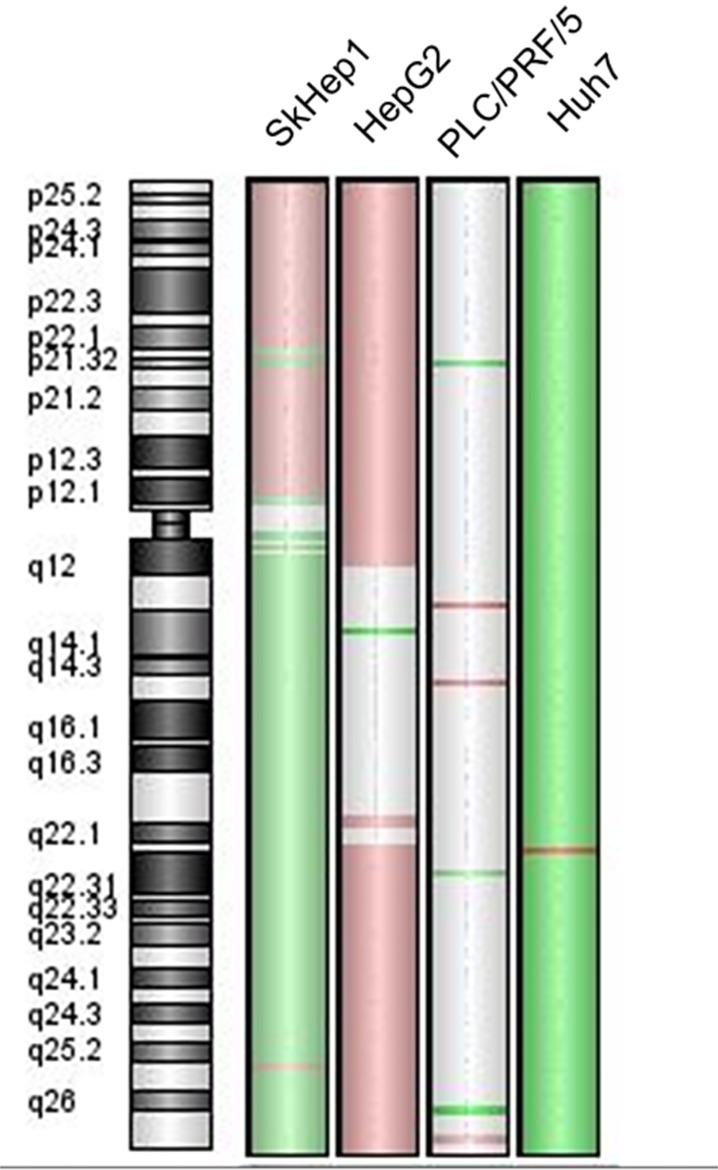
Chromosome 6 genomic profile of the cell-lines SkHep1, HepG2, PLC/PRF/5 and Huh7 The green colour on the chromosome ideogram indicates loss of the fragment, the red indicates gain and the grey indicates a balanced status.

Except for PLC/PRF/5 cells, all hepatoma cell-lines exhibited an aberrant genomic profile at 6q24.2, where the *PLAGL1* gene maps. Huh7 cells have losses of genetic material from almost the whole chromosome 6, but gains of the chromosome region 6q22.2. SkHep1 cells showed losses of genetic material from the long arm of chromosome 6, and a specific amplification of 6q25.2. These cells also have gains of the short arm of the chromosome 6, but with punctual deletions at 6p21.32 and 6p21.33. Regarding the region 6q24.2, the log-ratios for Huh7 and SKHep1 cells were −1.368 and −0.582, respectively, indicating that both tumor cell lines presented losses at the locus of *PLAGL1* gene. On the contrary, HepG2 cells exhibited gains of the short arm of chromosome 6, while the long arm presented a punctual loss at q14 and gains of the region q22-qter. This cell line showed positive values of logratio (≈0.500) of the probes used specifically for the fragment 6q24.2 where *PLAGL1* maps, thus indicating gain of material. Finally, the hepatoma cell line PLC/PRF/5, apart from few punctual gains and losses in other chromosomes, did not present changes at chromosome 6 (Figure 1 and Table 1).

**Table 1:** Genomic profile of the chromosome regions where the *PLAGL1*, *p53*, *PPARy* and *p21* genes map in four hepatoma cell-lines

Cell line	Gene			
	*PLAGL1*	*p53*	*PPARy*	*p21*
HepG2	0.507	0.000	0.000	0.517
SkHep1	−0.582	0.398	−0.571	0.277
Huh7	−1.368	0.000	0.000	−1.277
PLC/PRF/5	0.000	0.000	0.000	0.000

Positive values of the log-ratios indicate gain of genetic material, negatives indicate losses and 0.000 means absense of genomic abnormalities.

The genome regions where the *p53*, *PPARy* and *p21* genes map were also studied by aCGH in the four cell lines. The tumor suppressor gene *p53* maps on chromosome 17p13.1 and the analysis demonstrated that this region is amplified in SkHep1 (logratio value = 0.398), while in the other hepatoma cell-lines Huh7, HepG2 and PLC/PRF/5 no abnormalities were detected. Regarding the *PPARy* gene (3p25.2), HepG2, Huh7 and PLC/PRF/5 did not exhibit quantitative changes, but the analysis of SkHep1 revealed loss at this region (logratio value = −0.571). The data obtained for the region where the *p21* gene maps (6p21.31) are consistent with gains of genetic material in the HepG2 and SkHep1 cell-lines (logratios values = 0.517 and 0.277, respectively). In PLC/PRF/5 cells aberrations at 6p2 were not detected, while in Huh7 a logratio value of −1.277 indicates losses of this chromosome region (Table 1).

Out of 11 tissue-samples from patients with primary liver tumors, seven yielded quality DNA for aCGH. The analysis of quantitative genomic alterations revealed log-ratios compatible with losses at 6q24.2 (*PLAGL1*) in three of them: H008, H010 and H005 (Table 2). While all analyzed tumor samples did not present abnormalities on the chromosome regions where the *p21* and *p53* genes map.

**Table 2:** Summary of the aCGH and *in situ* PLAGL1 and p21 proteins expression data from primary liver tumors

Sample	Diagnostic	aCGH	Immunohistochemistry	
		*PLAGL1* Logratio	PLAGL1	p21
H001	HCC	0.000	−	−
H002	HCC	NA	+	−
H003	HCC	0.000	+	−
H004	HCC	NA	+++	++
H005	HCC	-0.472	+	−
H012	HCC	0.000	++	+
H013	HCC	NA	++	+
H008	FNH	-0.422	+	−
H009	CCC	0.000	++	+
H010	HAC	-0.605	++	++
H011	HAC	NA	+++	−
H015	Normal tissue	NA	+++	+++

The expression of PLAGL1 and p21 was evaluated in eleven tumor samples and one from normal liver tissue. Except two tumors, all exhibited lower level of PLAGL1, while expression of p21 in all of them was absent or lower than in normal liver tissue. HCC: hepatocellular carcinoma; FNH: focal nodular hyperplasia; CCC: cholangiocellular carcinoma; HAC: Hepatoadenocarcinoma. Negative values of the logratios indicate losses of genetic material, 0.000 means absense of genomic abnormalities and NA stands for not analyzed. Score of each marker was calculated as described in Materials and Methods: strong expression (+++), moderate expression (++), weak expression (+) and negative expression (−).

### Methyl specific PCR

**Figure 2:**
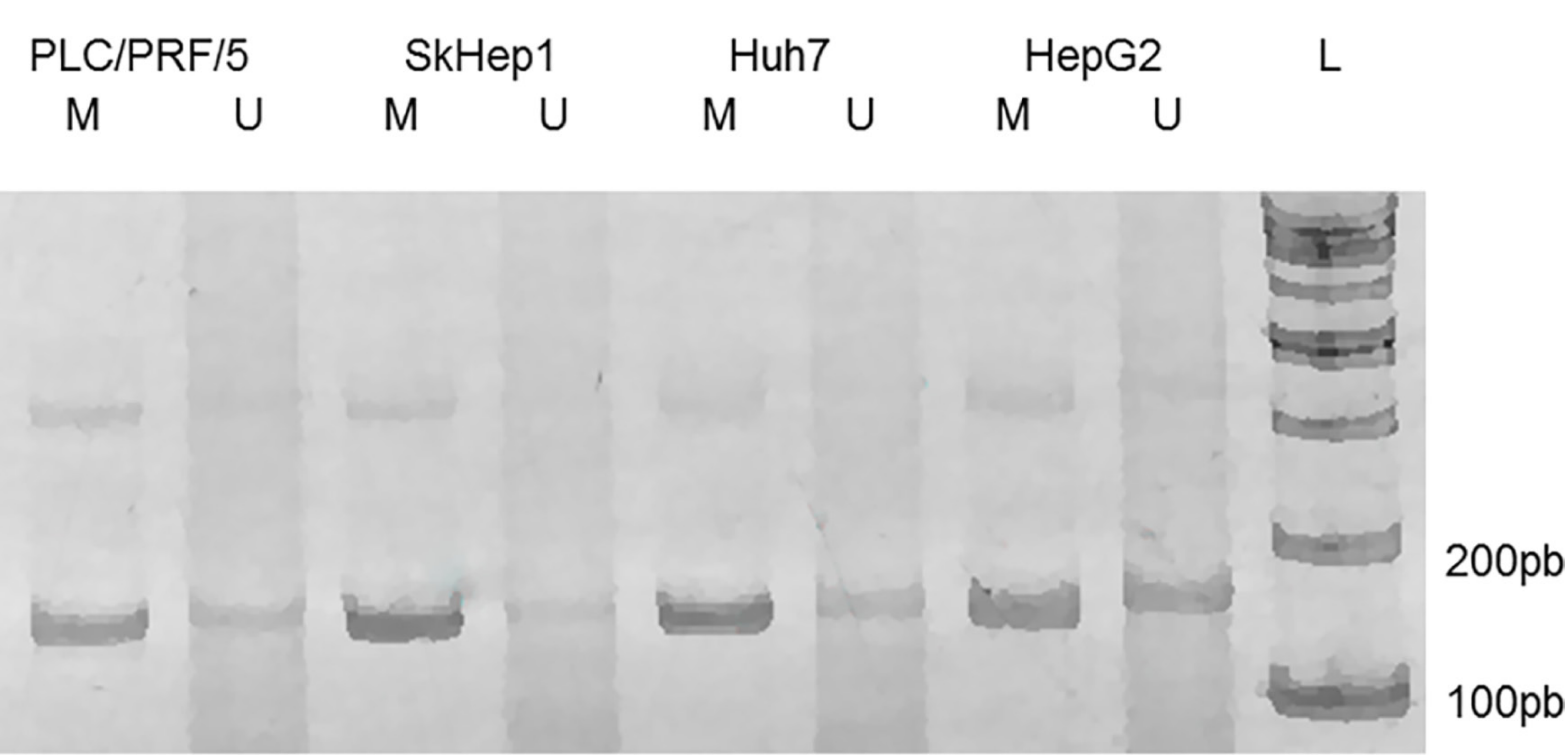
MS-PCR of the genomic region corresponding to the P1 promoter of *PLAGL1* gene Gel electrophoresis of the PCR products showed that the methylated DNA-fraction is larger than the unmethylated. L, size marker; M, methylated; U, unmethylated.

To study the methylation state of the P1 promoter of the *PLAGL1* gene we performed MS-PCR. All cell lines presented a fraction of DNA methylated and another unmethylated. However, conspicuous differences in terms of intensity of the bands corresponding to these fractions were observed after gel electrophoresis in three cell lines. HepG2 was the only cell line in which the DNA-band of methylated P1 promoter was rather similar to that of unmethylated promoter, whereas in the SkHep1, Huh7 and PLC/PRF/5 cell lines the methylated DNA fraction was much more intense than the unmethylated fractions (Figure 2).

### Proliferation assays and flow cytometry

**Figure 3:**
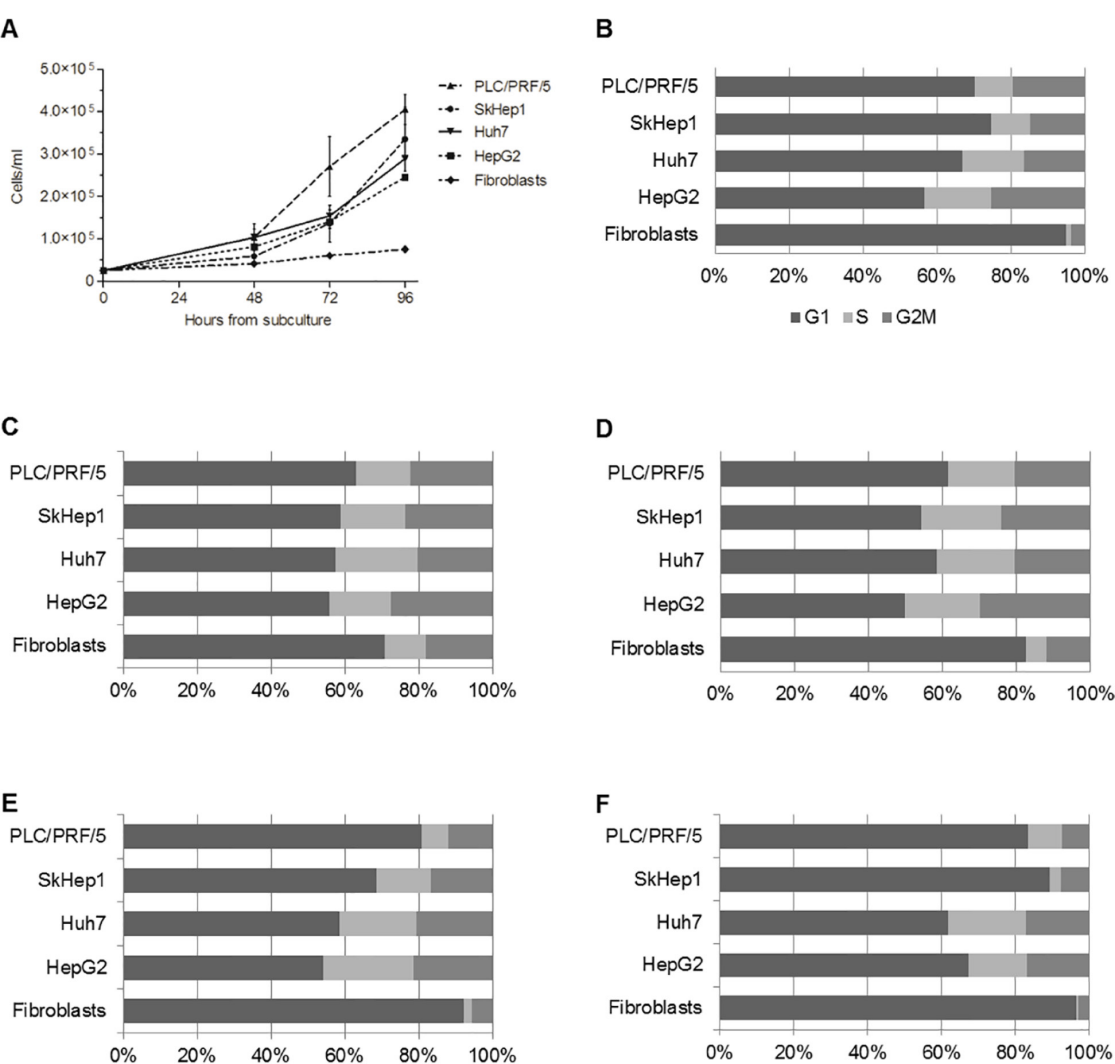
HepG2, Huh7, SkHep1 and PLC/PRF/5 cells proliferate faster than non-tumoral fibroblasts (**A**) Aliquots of 25 × 10^3^ cells/ml were seeded in 4 flasks and cultured in 10 ml of DMEM medium containing 10% FBS for 24, 48, 72 and 96 h. At each time point, the cells were harvested, and their number counted in a Neubauer chamber. Fibroblasts have a constant growth during the experiment and reached 7.5 × 10^4^ cells/ml at 96 h, in contrast to the 2.5–4.5 × 10^5^ cells/ml reached by HCC cells at the same time point. (**B**–**F**) Show the percentage of cells in each phase of the cell cycle at 0 h, 24 h, 48 h, 72 h and 96 h, respectively.

To estimate the proliferation capacity of the cell-lines, the number of cells at different time points during culturing was determined, and used to calculate the doubling time (DT). Among tumor cell-lines, PLC/PRF/5 cells were the most prolifertive; our DT calculation showed that these cells duplicated their number after 23 h of culture. SkHep1 cells were also highly proliferative (DT = 26 h), both cell-lines reached confluence faster than HepG2 and Huh7 whose DT were 29 h and 27 h, respectively. Fibroblasts had low proliferation capacity compared to tumor cells, and this is reflected by the high DT (59 h) (Figure 3A). To further study the proliferation capacity of the tumor cells, the amount of cells in the different phases of the cell cycle were determined by flow cytometry. The percentage of cells in the G2/M phase of the cell cycle increased from the beginning of the experiment (T = 0 h) until 48 h in culture, and from this time-point forward the fraction of cells in this stage decreased in all cell-lines. However, this analysis showed that during the entire assay the tumor cell-lines always presented higher percentage of cells in the G2/M phase than non-tumoral fibroblasts. Accordingly, the amount of fibroblasts in G1 phase was always higher than tumor cells (Figure 3B–3F).

### *PLAGL1*, *p53* and *PPARy* transcription

**Figure 4:**
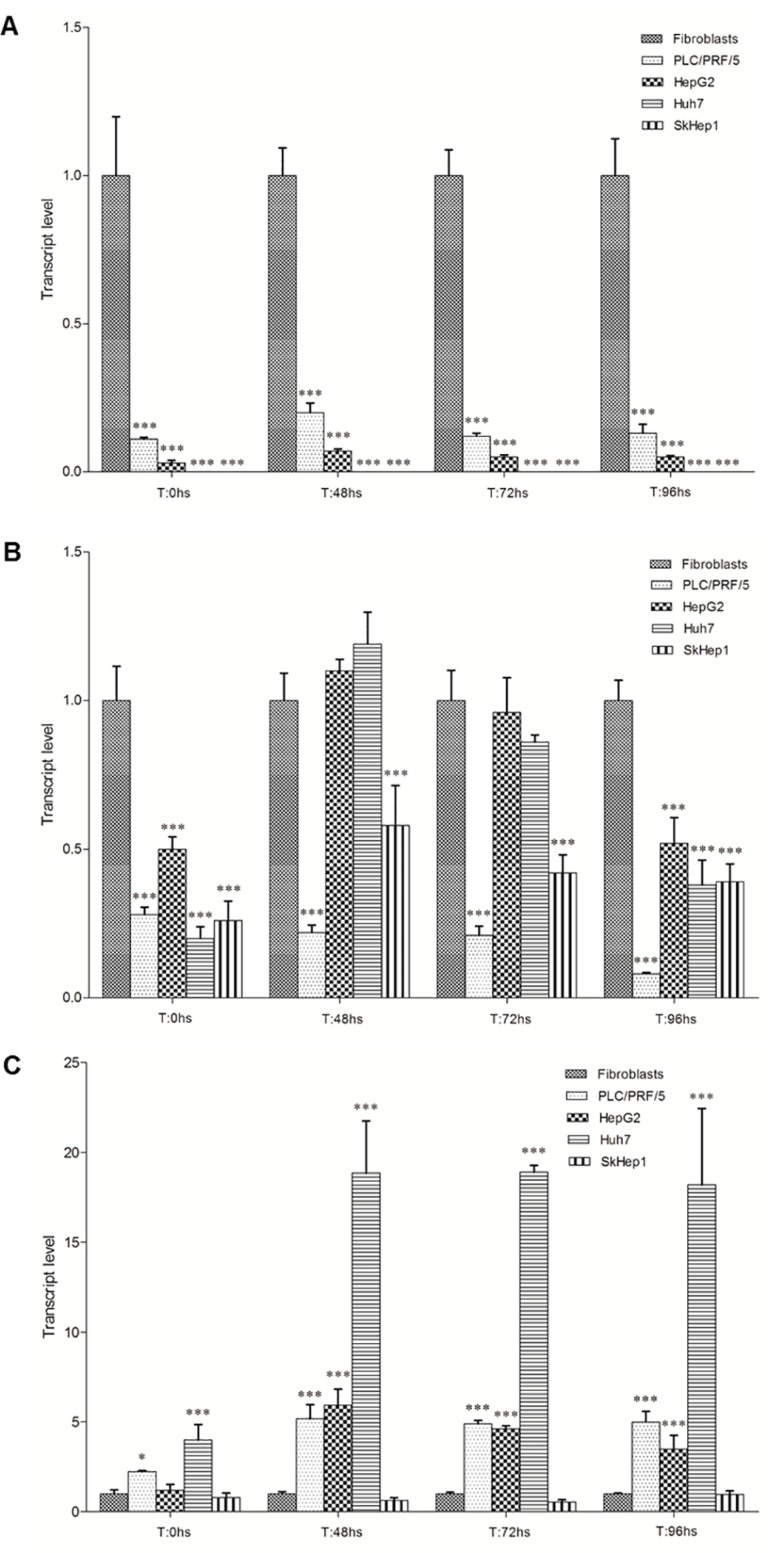
Transcript level of *PLAGL1*, *p53* and *PPARy* during proliferation The mRNA level of each tumor cell-line was compared with that of fibroblasts at the same time point of the proliferation curve. (**A**) *PLAGL1*. (**B**) *p53*. (**C**) *PPARy*. Bars represent mean ± SD. Asterisks indicate that the mRNA levels compared are significantly different; ^*^p < 0.05, ^**^p < 0.001, ^***^p < 0.0001.

mRNA levels of the *PLAGL1*, *p53* and *PPARy* genes in all cell types and at the different time points during proliferation were estimated by quantitative RT-PCR, and expressed respect to the level of the *PPIA* gene. Then we compared the transcript levels of *PLAGL1*, *p53* and *PPARy* of each tumor cell line with those obtained for fibroblasts at the same time point of the proliferation curve. This study showed that *PLAGL1* and *p53* transcript levels were in general significantly lower in all HCC cell-lines compared to fibroblasts, except for *p53* transcript level in HepG2 and Huh7 cells at 48 h and 72 h (Figure 4A and 4B). Statistics confirmed that *PLAGL1* transcript level of tumor cells was significantly lower than that of non-tumor cells (*p* < 0.05) (Figure 4A). This study also showed that *PLAGL1* mRNA levels in SkHep1 and Huh7 were insignificant (expression value = 0.00) respect to fibroblasts (Figure 4A). On the contrary, the levels of *PPARy* transcripts were in general higher in HCC cells than in fibroblasts during proliferation. Moreover, this gene transcription was exceedingly higher in Huh7 cells than in the other tumor cell-lines (Figure 4C).

**Figure 5:**
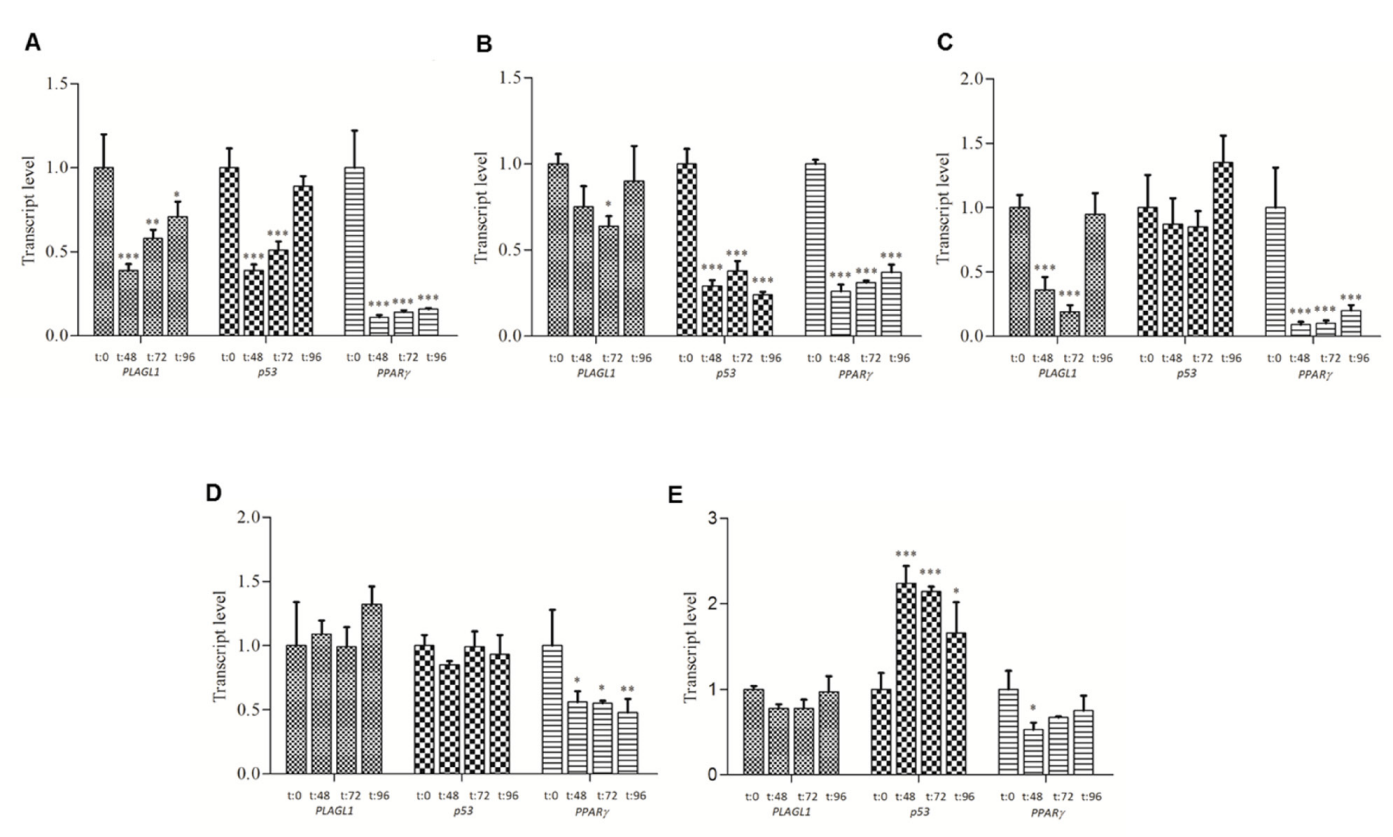
*PLAGL1*, *p53* and *PPARy* transcript level during *in vitro* cell growth (**A**) Fibroblasts. (**B**) PLC/PRF/5. (**C**) SkHep1. (**D**) HepG2. (**E**) Huh7. The statistical comparisons were made between any given time point and T = 0 h for each cell line individually. Bars represent mean ± SD. Asterisks indicate that the difference is significant; ^*^p < 0.05, ^**^p < 0.001, ^***^p < 0.0001.

qPCR data was also utilized to perform a detailed analysis of the dynamic of *PLAGL1*, *p53* and *PPARy* genes transcription in each cell line during proliferation. In fibroblasts, *PLAGL1* mRNA level decreased after the release from serum starvation until T = 48 h and then gradually increased until the end of the experiment (T = 96 h) (Figure 5A). Among the tumor cell-lines, only SKHep1 cells exhibited a transcriptional profile similar to that of fibroblasts, i.e., a significant reduction of the *PLAGL1* mRNA level until T = 72 h and then increased to levels statistically not different to that obtained at T = 0 h (Figure 5C). Whereas PLC/PRF/5, HepG2 and Huh7 cells did not show variations in the transcript levels of *PLAGL1* gene along the growth curves (Figure 5B, 5D and 5E).

The PLAGL1 protein interacts, among others, with p53 for controlling cell proliferation [13]. Therefore, we wondered whether transcription of the genes encoding these two proteins would keep any relationship with each other. As mentioned before, we found that *p53* mRNA level in fibroblasts decreased from T = 0 h until T = 48 h during the proliferation assay, and then gradually increased until T = 96 h, following a similar transcription pattern of *PLAGL1* in this cell type (Figure 5A). Tumor cells presented different dynamics of this gene transcription along the proliferation curve. In PLC/PRF/5 cells *p53* gene transcript level decreased significantly after the release from serum starvation and remained at low levels during the proliferation assay (Figure 5B). In SkHep1 and HepG2 cells *p53* transcription remained without changes during the proliferation experiment (Figure 5C and 5D). Huh7 cells, on the contrary, exhibited a significant, and sustained, increase in the level of *p53* gene transcription respect to the mRNA level that this cell type had at T = 0 h of the experiment (Figure 5E).

Considering that PLAGL1 acts also as a transcription factor of the *PPARy* gene, RT-qPCR was performed to determine the transcript level of this gene during cell proliferation. This analysis revealed that in all cell lines the transcript level of *PPARy* decreased respect to the level at T = 0 h, and it remained low during the proliferation experiment (Figure 5).

### PLAGL1, p53 and p21 proteins expression

**Figure 6:**
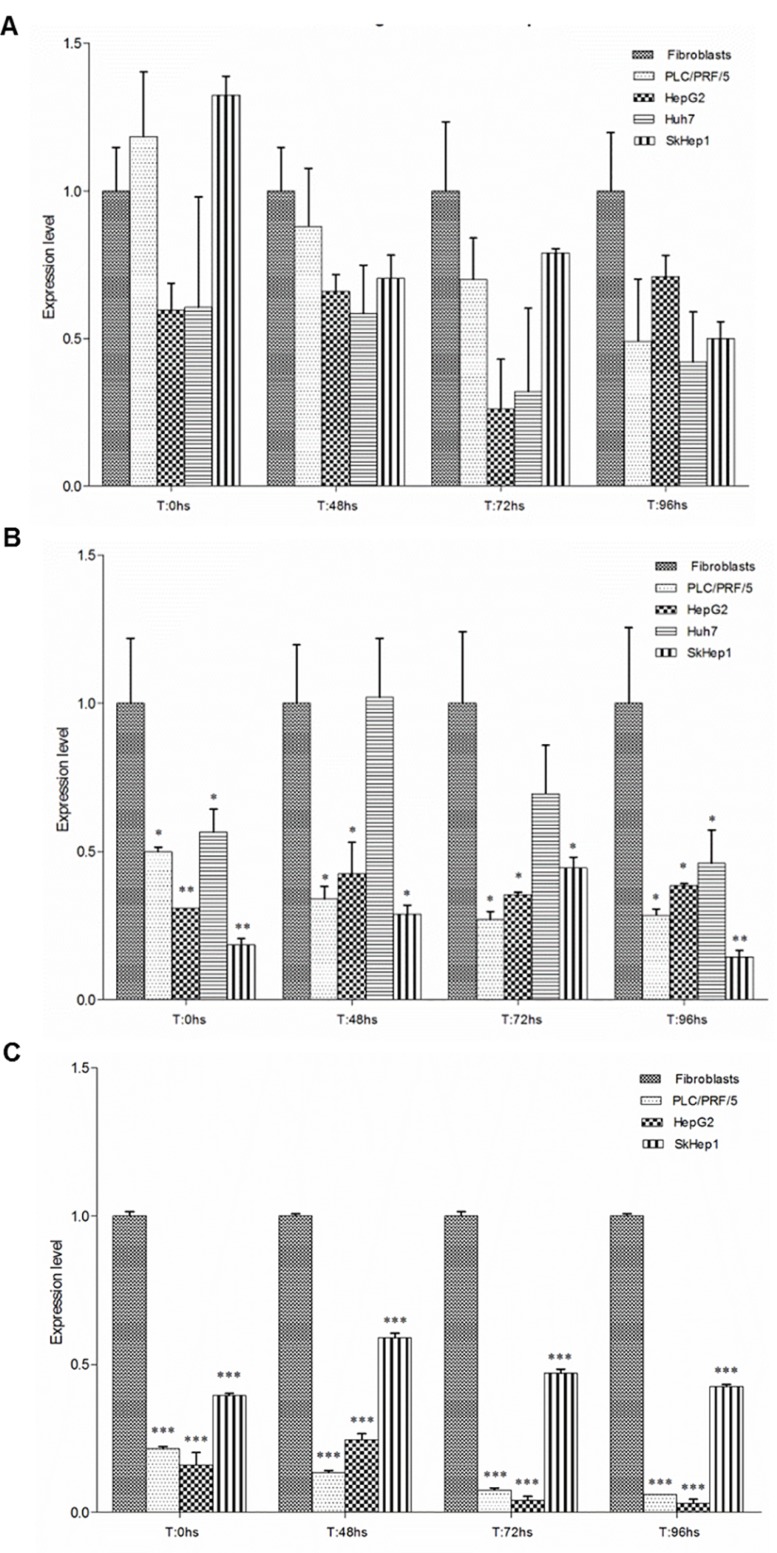
Proteins expression in normal and tumor cells during proliferation PLAGL1 (**A**), p53 (**B**) and p21 (**C**) proteins levels were determined, and compared with that of fibroblasts at each time point. Bars represent mean ± SD. Asterisks indicate statistically significant differences; ^*^p < 0.05, ^**^p < 0.001, ^***^p < 0.0001.

**Figure 7:**
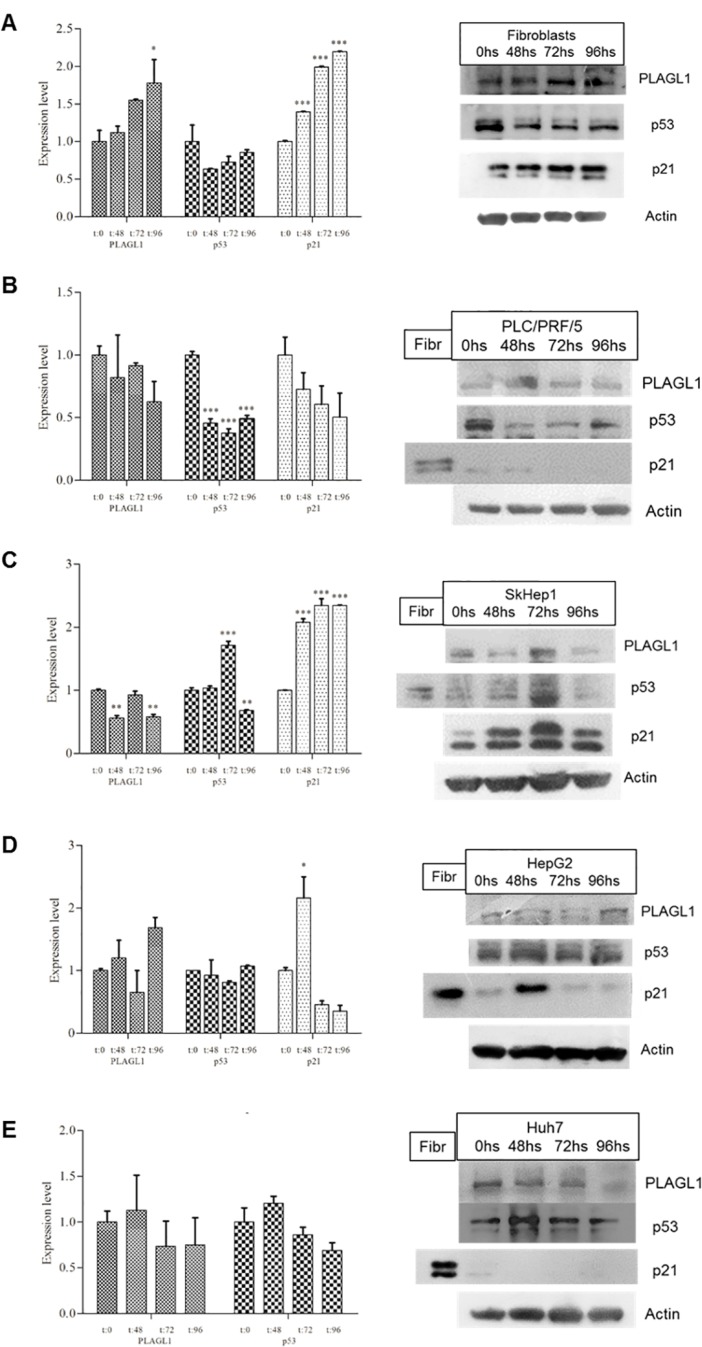
Expressiont level of PLAGL1, p53 and p21 of each cell line during proliferation The Western blot data corresponding to each time point was compared with that of T = 0 h. (**A**) Fibroblasts. (**B**) PLC/PRF/5. (**C**) SkHep1. (**D**) HepG2. (**E**) Huh7. A protein extract from fibroblasts (Fibr) was used as a signal control for the antibody p21. Representative blots are shown at the side of each graph. Actin was used as loading control. Bars represent mean ± SD. Asterisks indicate statistically significant differences; ^*^p < 0.05, ^**^p < 0.001, ^***^p < 0.0001.

The expression levels of PLAGL1, p53 and p21 proteins were determined in all cell-lines by Western blot analysis. We compared the data of PLAGL1, p53 and p21 protein levels for each tumor cell-line with those obtained for fibroblasts at the same time point of the proliferation curve. Despite high variability in PLAGL1 expression was seen, the statistical analysis demonstrated that there were not significant differences in the expression level of PLAGL1 between tumor cells and fibroblasts (Figure 6A). Regarding to p53 protein, the comparisons showed that the expression level of this protein was significantly lower in tumor cell lines than in fibroblasts, except for Huh7 cells after 48 h and 72 h in culture (Figure 6B). Similar analysis of the data obtained for p21 protein revealed that its expression level was significantly higher in fibroblasts than in tumor cell lines along the experiment (Figure 6C). The data was also analyzed for each cell-line individually. In fibroblasts, PLAGL1 and p53 protein levels did not experiment demonstrable changes during cell growth, except for PLAGL1 protein that exhibited a significant increase just at the end of the proliferation curve (T = 96 h) (Figure 7A). This is in clear contrast with the dynamics of *PLAGL1* and *p53* genes transcription, which experimented a significant reduction in their transcripts level after serum starvation and then increasing until T = 96 h (Figure 5A). The expression of p21 protein exhibited a different profile; statistically significant increases were detected at each time-point during fibroblasts proliferation (Figure 7A).

The tumor cell-lines showed a rather uniform expression level of PLAGL1 during the growth assays, except for SkHep1 cells that presented significant lower expression levels at 48 and 96 h of the experiment (Figure 7). As to the expression of p53 and p21 proteins, we found different profiles among tumor cell-lines. In PLC/PRF/5 cells, these proteins levels experimented a reduction respect the values obtained at 0 h, but only the reduction of p53 expression reach statistically significant levels during proliferation (Figure 7B). In SkHep1 cells, p53 expression increased at 72 h and decreased at 96 h, while p21 expression doubled the level during proliferation (Figure 7C). The HepG2 cell-line presented similar levels of p53 protein expression in all the time-points of the proliferation curve, whereas p21 protein underwent a conspicuous increase of expression at 48 h and then returned to similar levels of that of the synchronized cells (Figure 7D). Finally, in Huh7 cells p53 protein expression level did not change along the experiment, and the p21 protein was not detected by Western blot (Figure 7E).

### PLAGL1 and p21 proteins expression *in situ*

**Figure 8:**
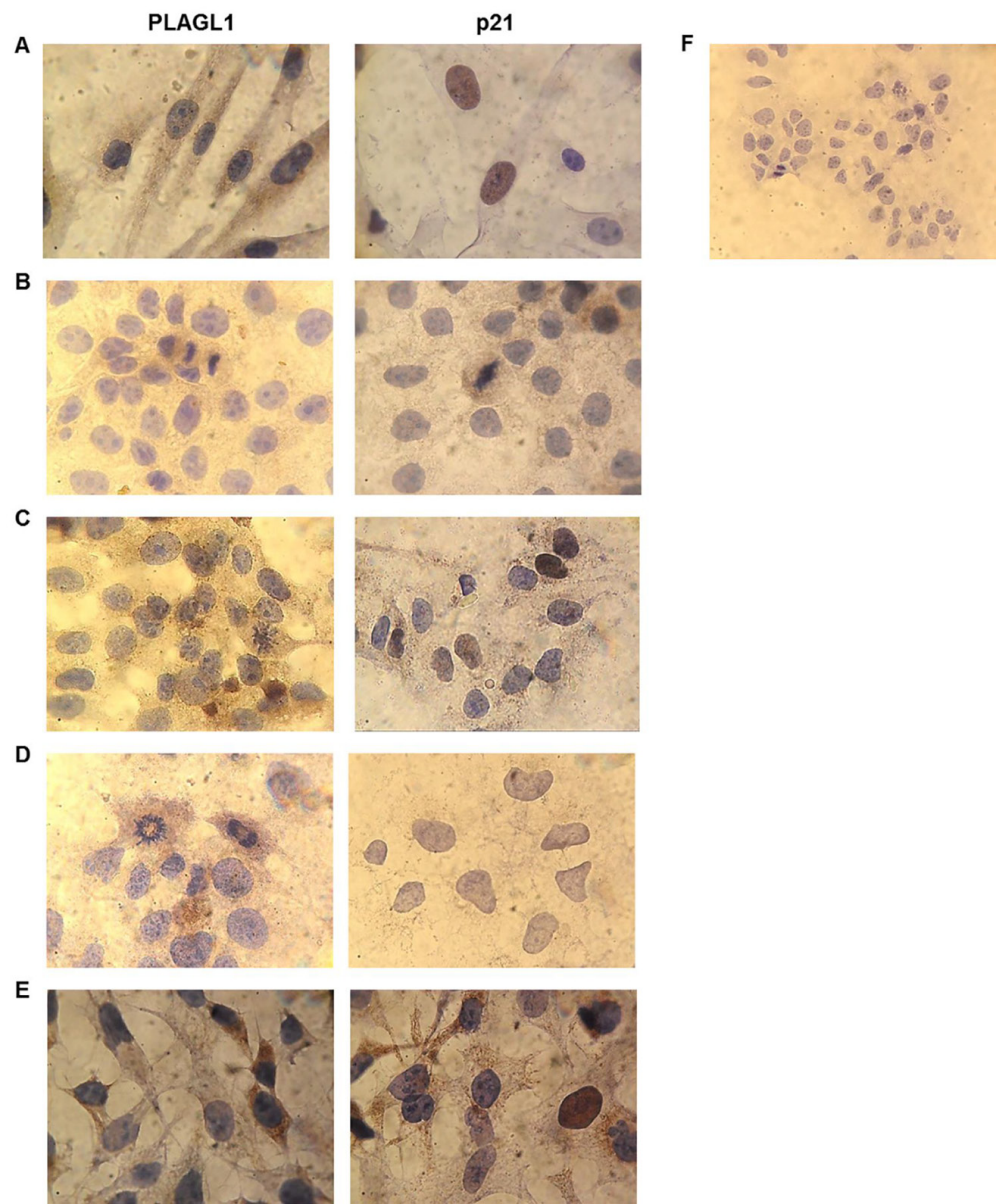
Immunocytochemistry for PLAGL1 and p21 proteins in tumor and normal cells Microscopic exam of the preparations revealed that PLAGL1 protein localized only in the cytoplasm of tumor cells, while p21 exhibited both nuclear and cytoplasmatic localization. Similar to Western blot analysis, this protein was not detected in Huh7 cells. (**A**) Fibroblasts; (**B**) PLC/PRF/5; (**C**) HepG2; (**D**) Huh7; (**E**) SkHep1; (**F**) Negative control. Magnification 1000× (A–E) and 400× (F).

The expression *in situ* of PLAGL1 and p21 proteins was determined by immunocytochemistry in the four hepatoma cell-lines and fibroblasts. PLAGL1 immunoreactivity was weakly detected in the cytoplasm of tumor cells, while in normal fibroblasts its expression was readily observed in the nucleus and the cytoplasm. As regard to p21 protein, nuclear immunoreactivity was detected in normal fibroblasts and in HepG2 and SkHep1 cells. While PLC/PRF/5 cells presented weak immunoreaction only in the cytoplasm, and it was not detected in Huh7 (Figure 8).

**Figure 9:**
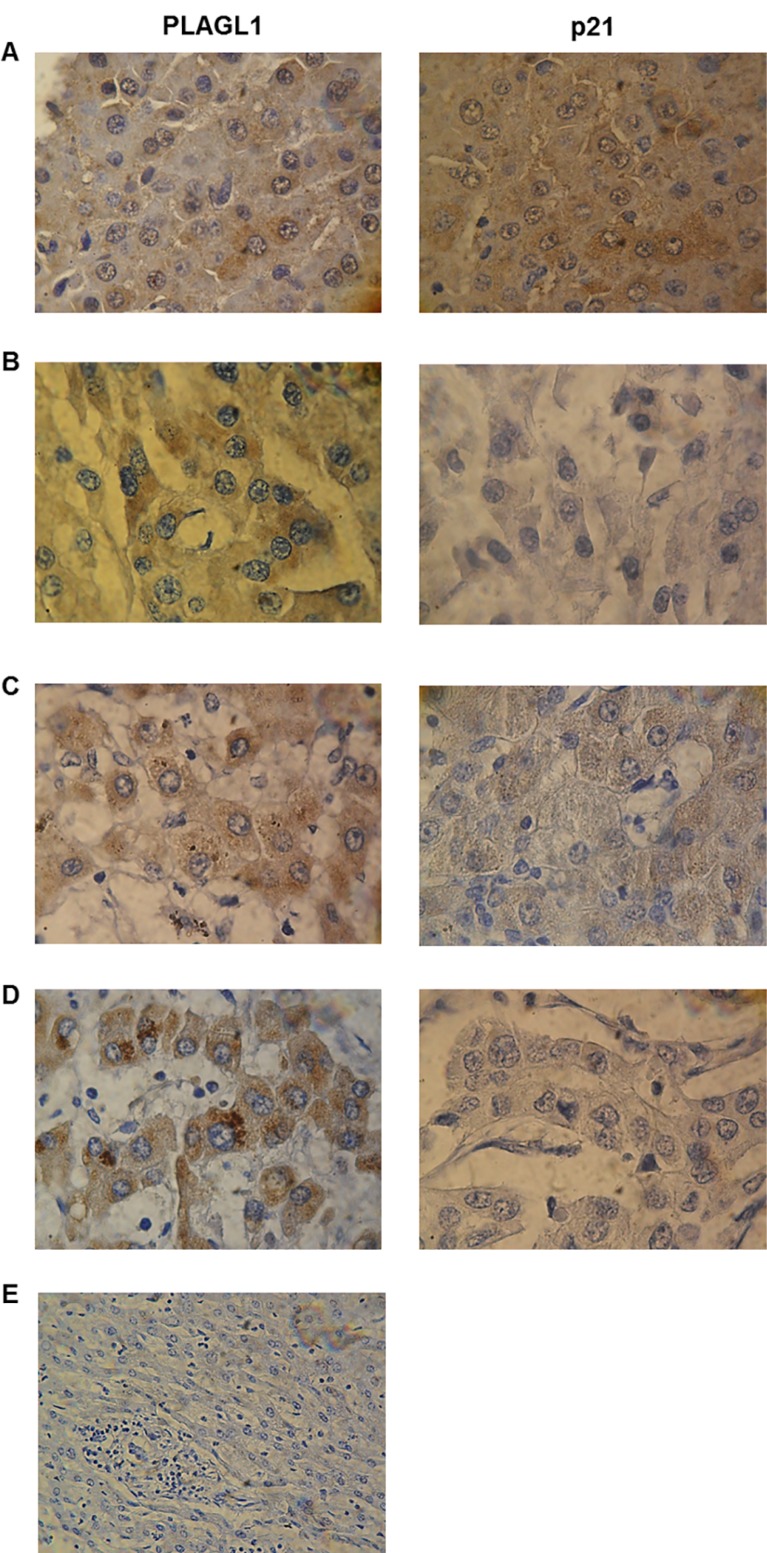
Immunohistochemical analyses of PLAGL1 and p21 proteins expression in liver tumors Representative photomicrograph of non-tumoral liver tissue stained with anti-PLAGL1 and anti-p21 antibodies (**A**). (**B**) H003. (**C**) H012. (**D**) H011. (**E**) Negative control. Weak or moderate expression of PLAGL1 and p21 proteins was observed in tumor samples compared to normal liver tissue. Note that both proteins were detected in the cytoplasm and nuclei of non-tumor liver, while PLAGL1 localized only in the cytoplasms of tumor cells. Magnification 1000× (A–D) and 400× (E).

PLAGL1 and p21 proteins expression *in situ* was also assessed in ten tissue samples from patients with hepatic tumors by immunohistochemistry. The microscopic analysis revealed that, except for two (H004 and H011), all tumor samples had lower expression of PLAGL1 protein than normal liver cells (Figure 9; Table 2). Moreover, we observed that the protein localized only in the cytoplasms of tumor cells, and that in one tumor (H011) the protein formed aggregated close to the nuclear membrane (Figure 9). As to the p21 protein expression, the microscopic exam revealed that in six out of the 11 samples the protein was not detected, and the remaining had lower level of p21 expression than non-tumoral cells (Table 2).

### PLAGL1 protein overexpression

**Figure 10:**
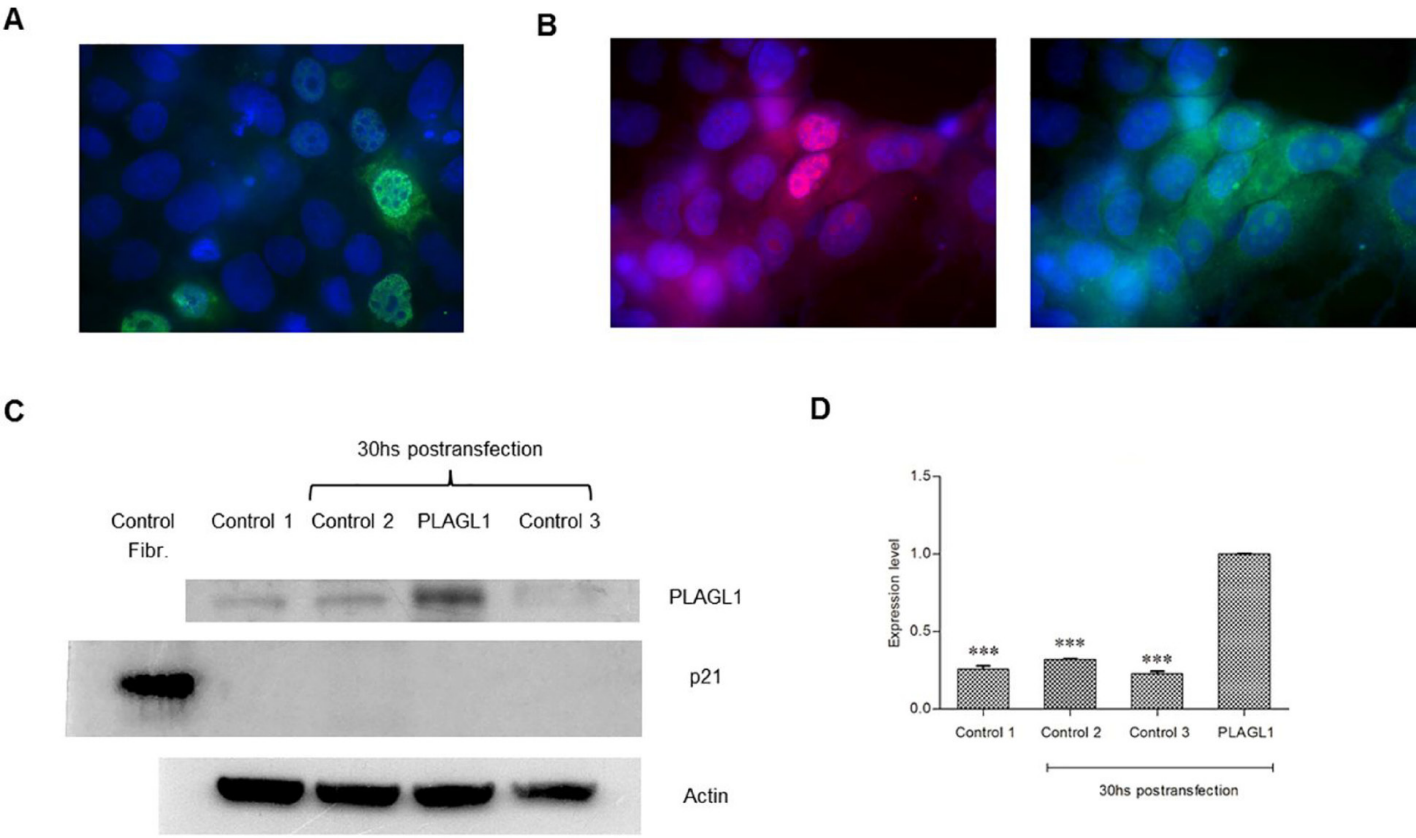
Overexpression of PLAGL1 protein in PLC/PRF/5 cells (**A**) Immunofluorescence of transfected cells with an antibody anti-PLAGL1(Green). (**B**) Immunofluorescence of transfected cells with antibodies against PLAGL1 (Red) and p21 (Green). (**C**) Representative blots of PLAGL1 and p21 proteins. A protein extract from fibroblasts (Fibr.) was used as a signal control for the antibody against p21. Actin was used as loading control. (**D**) PLAGL1 relative expression: Control 1 = T: 0 hs; Control 2 = No plasmid; Control 3 = Empty plasmid; PLAGL1 = PLC/PRF/5 cells transfected with the plasmid prk7-PLAGL1. Bars represent mean ± SD. Asterisks indicate statistically significant differences (^***^p < 0.0001).

**Figure 11:**
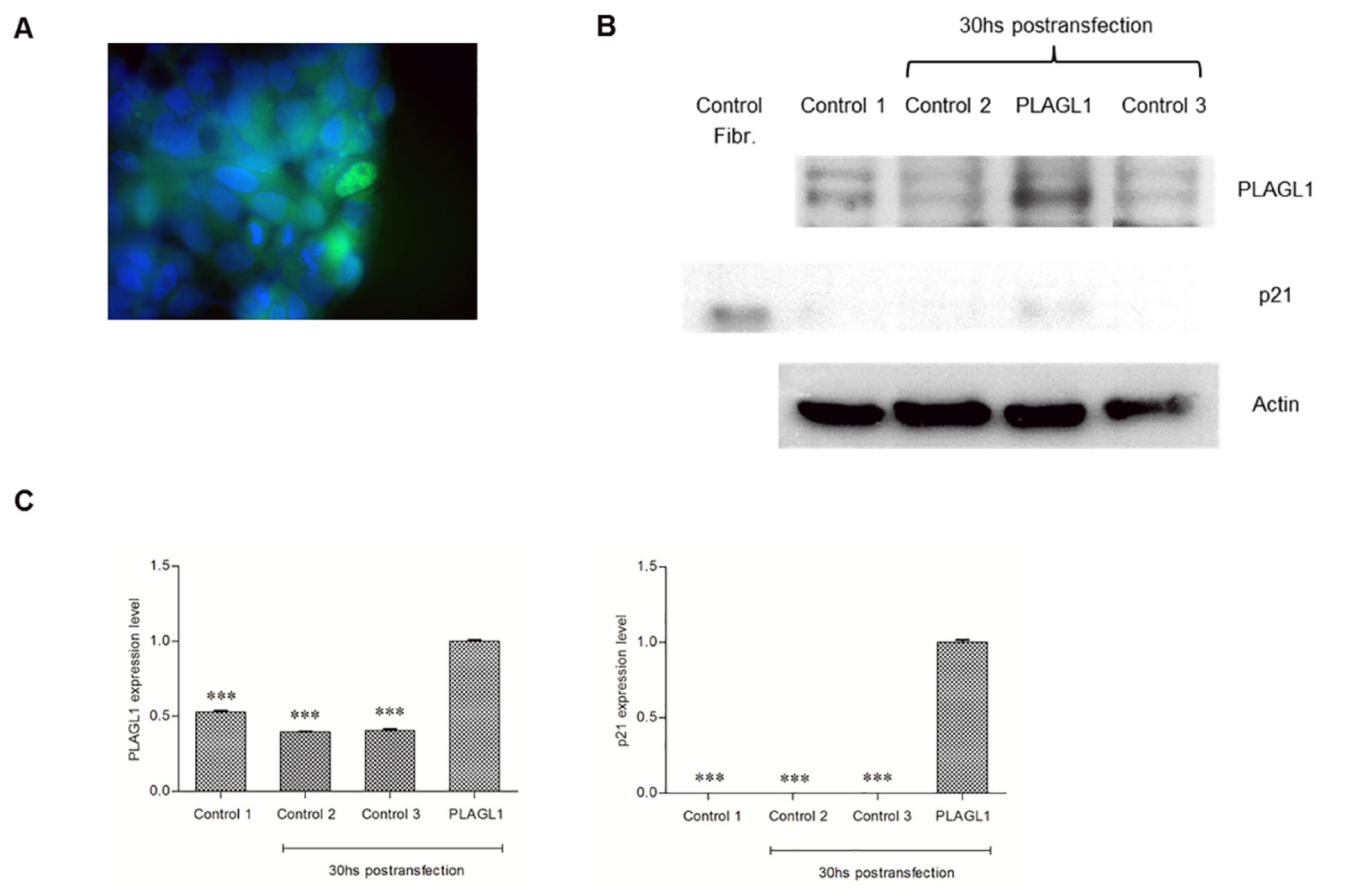
Overexpression of PLAGL1 protein in HepG2 cells (**A**) Immunofluorescence of transfected cells with antibody anti-PLAGL1 (green). (**B**) Representative blots of PLAGL1 and p21 proteins. A protein extract from fibroblasts (Fibr.) was used as a signal control for the antibody p21. Actin was used as loading control. (**C**) PLAGL1 and p21 relative expression: Control 1 = T: 0 hs; Control 2 = No plasmid; Control 3 = Empty plasmid; PLAGL1 = HepG2 cells transfected with the plasmid prk7-PLAGL1. Bars represent mean ± SD. Asterisks indicate statistically significant differences (^***^p < 0.0001).

To study the effect of PLAGL1 overexpression on HCC cells proliferation, we performed transfection assays in two tumor cell-lines (PLC/PRF/5 and HepG2) with a plasmid encoding the full-length protein. Despite of the low efficiency of the transfection method (about 10% in both cell types), the PLAGL1 protein level increased significantly in cell transfected with prk7-PLAGL1, respect to non-transfected cells (Figures 10 and 11). However, Western blot analyses performed to determine the p21 protein level revealed that it increased, in response to PLAGL1 overexpression, only in HepG2 cells (Figures 11B, 11C), which harbour a wild type *p53* gene. Simultaneously we investigated whether this overexpression of PLAGL1 protein affects proliferation of tumor cells, by determining the number of cells for each experimental condition after 30 hs post-transfection. We found that PLC/PRF/5 cells without transfection proliferated actively from 2.2 × 10^5^ cell/ml at T = 0 hs to 4.2 × 10^5^ cell/ml at T = 30 hs, while cells transfected with prk7-PLAGL1 reached 3.2 × 10^5^ cell/ml at T = 30 hs. As mentioned before, HepG2 cells were the least proliferative, and this was confirmed with this experiment. Transfection of these cells with the prk7-PLAGL1 construct almost arrested cell growth (1.70 × 10^5^ cell/ml at T = 0 hs vs 1.75 × 10^5^ cell/ml at T = 30 hs), compared with untransfected cells (2.5 × 10^5^ cell/ml at T = 0 hs).

## DISCUSSION

*PLAGL1* is a TSG involved in the pathogenesis of several tumor types, including ovarian, gastric, pituitary and colorectal cancer [20, 24, 25, 30, 31]. Considering that LOH at the *PLAGL1* locus and hypermethylation of its P1 promoter occur more frequently than gene mutations, the mechanisms by which this gene participates in the tumorigenic process seem to be different from those of typical TSGs. In fact, punctual mutations of the *PLAGL1* gene were detected in only 71 (0.3%) out of 21,029 tumor samples of all kind registered in the Catalog of Somatic Mutations in Cancer (COSMIC; http://cancer.sanger.ac.uk/cosmic). To add more complexity, it has been also reported that overexpression of *PLAGL1* was detected in some human neoplasms such as glioma and clear cell renal cell carcinoma, suggesting an oncogenic function, as well [28, 29]. As regard to HCC, Midorikawa *et al.* found that LOH at the chromosome region 6q24, hypermethylation of *PLAGL1* promoter at the remaining allele and low mRNA levels were frequent in tissue samples from patients with hepatoma [26]. Therefore, we wondered how genomic imbalances at the chromosome region 6q24, where *PLAGL1* gene maps, would affect the transcription and expression level of this gene, and ultimately how this influences the proliferative capacity of liver cancer cells. To this aim, we investigated the profile of 6q24 aberrations of the hepatoma cell lines HepG2, Huh7, PLC/PRF/5 and SkHep1. The aCGH analysis showed that Huh7 and SkHep1 cells present losses of genetic material at 6q24.2, HepG2 exhibited gains, and PLC/PRF/5 has no abnormalities in this region (Figure 1 and Table 1). Also three out of seven liver tumors we analyzed had losses of genetic material from this region. This data may suggest that abnormalities at this chromosome region are common in HCC, however it should be kept in mind that other regions, such as 1p, 8p and 17p, are affected by imbalances more frequently [26, 32]. Moreover, these changes are not specific of this tumor type, losses at chromosome 6q have also been found, among others, in pheochromocytomas and gastric adenocarcinomas [31, 33]. We also determined that the regulator region of *PLAGL1* (P1 promoter) is heavily methylated in the four hepatoma cell lines (Figure 2), and most likely this explain the low mRNA level of the *PLAGL1* gene in these tumor cell lines (Figure 4A). Reduced transcription, due to hypermethylation of P1 promoter of this gene, was already demonstrated in gastric adenocarcinomas and HCC [26, 31]. However, the relationship between genomic imbalances, the methylation status of the P1 promoter and the transcription level of this gene is apparently not direct. In fact, the tumor cell-line HepG2 presents gain of genomic material at 6q24 (Figure 1), the lowest degree of P1 promoter methylation (Figure 2), and still the transcript and expression level of *PLAGL1* is similarly low to that of the other hepatoma cell lines (Figure 4A and Figure 6A).

The proliferation assays we performed by cell-counting demonstrated that the HCC cells grow much faster than the normal fibroblasts used as control. Accordingly, flow cytometry analysis showed that during the entire assay, the four tumor cell lines always presented higher percentage of cells in the G2/M phase than non-tumoral fibroblasts. This results were somehow expected, since cell cycle deregulation, which results in unscheduled proliferation, is a common feature of human cancer. In this context we determined the mRNA and protein levels of *PLAGL1*, and the RT-qPCR analysis revealed that transcription of *PLAGL1* in tumoral cells is significantly lower than in normal fibroblasts, but no significant differences in terms of protein expression were detected between tumor cell-lines and normal fibroblasts. As mentioned before, reduced transcription of *PLAGL1* is common to several tumor types [24, 25, 31], but the results of protein expression are intriguing. It is well accepted that the abundance of proteins may or may not keep a direct relationship with the mRNA levels because of the differences in the mechanisms regulating transcription and translation, and also that mRNAs are produced at a much lower rate, and are less stable than proteins [34], inclusive specific PLAGL1-targeting miRNAs (miR-98; miR-15a/16; miR-23a/b), leading to mRNA degradation, have been identified [35, 36]. Also it has to be taken into consideration the cellular organization of *PLAGL1* gene transcription. Royo *et al.* demonstrated that *PLAGL1* mRNA accumulates juxtaposed to the nucleolus in normal fibroblasts, and that it is released from this nuclear compartment, increasing the synthesis of PLAGL1 protein, in response to stimuli that induce cell cycle arrest [37]. Thus the silmilar protein level between these two cell types is perhaps the result of less pronounced nucleolar retention of *PLAGL1* transcripts in tumor cells than in normal fibroblasts. An alternative explanation may be that Western blot data is not appropriated for comparing the abundance of the PLAGL1 protein between different cell types, despite the growth conditions were identical. This seems to be supported by our findings from immunocytochemistry analyses, which indeed showed differences in terms of *in situ* expression of PLAGL1 protein in tumor cells (lower) compared to normal fibroblast (Figures 8 and 9), suggesting that the level of PLAGL1 protein needed to control the proliferation of normal cells is different from that for tumor cells. Immunocytochemistry also revealed that the signal was only present in the cytoplasm of hepatoma cells, and this is in clear conflict with its nuclear functions as a transcription factor and cofactor of other proteins [38]. Alterations in the nuclear localization signal (NLS) domain of the protein may not be responsible for this cellular localization of PLAGL1, since mutations of this gene are uncommon in tumors (COSMIC; http://cancer.sanger.ac.uk/cosmic). Instead, it is tempting to speculate that the defect may be due to abnormalities in other players of the cellular trafficking, such us Importinα1 (*KPNA2*) or Exportin1 (*XPO1*) [38, 39].

As mentioned before, PLAGL1 interacts with p53, and then the complex induces the expression of the *p21* gene, which is essential for controlling proliferation through cell cycle arrest [14, 16]. Therefore, we also profiled the genomic regions where these genes map. aCGH revealed that, except for SkHep1, the tumor cell lines do not harbour quantitative imbalances in the *p53* region (Table 1). PLAGL1 and p53 protein expression usually remained uniform during tumor-cells proliferation (Figure 7), but almost always at lower levels in HCC cells than normal fibroblasts. RT-qPCR studies demonstrated that *p53* transcription is also rather constant during HCC cells proliferation, and this is in clear contrast with the dynamic transcription observed in normal fibroblasts (Figure 5). This cell-type exhibited the highest level of *p53* mRNA at T: 0 h, which decreased to a minimum at 48 h in culture and then increased again until the end of the experiment (T:96 h), similar to the transcription profile showed by *PLAGL1* gene (Figure 5A), and it was accompanied by a reduction of the percentage of cells in G2/M phase conforming cell-cycle regulation takes place. Huang *et al.* demonstrated the interaction between PLAGL1 and p53 proteins, and proposed that both proteins participate together in the control of normal growth [14]. While Rozenfeld-Granot *et al.* reported that p53 acts as a transcription factor of *PLAGL1* gene [40]. Now our study provides evidence for an apparent orchestrated transcription process of both genes during normal cells proliferation, which gets disturbed in HCC cells. The increase in the transcriptional activity may serve for maintaining constant the level of protein needed for cell-cycle regulation. Supportive evidence comes from our PLAGL1 overexpression studies. Transfection assays in PLC/PRF/5 and HepG2 cell-lines, with a construct that encodes the full-length protein, demonstrated that PLAGL1 level increased in cell transfected with prk7-PLAGL1, respect to non-transfected cells (Figures 10 and 11), and that the level of p21 protein increased only in HepG2, the cell-system with functional *p53* gene. In spite of the fact that statistics showed that the differences were not significant, the data from cell count indicates that cell-growth lessened in both cell-types. Obviously, caution is warranted when interpreting these findings, but them suggest that abrogation of PLAGL1 function plays a role in HCC cells proliferation, like in other tumor types [20, 25, 31]. Moreover, one has to accommodate the fact that that PLC/PRF/5 cells with non-functional p53 also underwent growth retardation, which indicates that PLAGL1 anti-proliferative activity is performed through additional mechanisms to those involving p53 and p21 proteins. In fact, Varrault *et al.* have recently shown that PLAGL1 target genes include numerous genes involved in extracellular matrix composition, cell adhesion and cell signalling [41].

The genomic study revealed that the region 6p21.3, where *p21* maps, showed gains of genetic material in SkHep1and HepG2, and losses in Huh7 (Table 1). Earlier studies performed by Koga *et al.* demonstrated, like us, that Huh7 cells have not detectable p21 expression (Figure 7E) [42], and most probably these results are consequence of the chromosome alteration in this cell line. The gain of genomic material in SkHep1 and HepG resulted in higher expression level of p21 in these cell-lines respect to PLC/PRF/5 cells, which does not harbour changes at 6p21.3 (Figure 1 and Table 1). However, statistical comparisons demonstrated that it is always lower than that of normal fibroblasts. This is in agreement with the data of immunocytochemistry analyses, which showed that the protein is hardly detected in the cytoplasm of tumor cells (Figure 8). The data of reduced expression of p21 during proliferation in HCC cell lines respect to non-tumoral cells, coinciding with low *p53* mRNA and protein level, fit well with the antiproliferative function of these proteins [43–46]. However, dysregulation of the balance between proliferation and cell death cannot be explained only by a decrease in the expression level of a few proteins. Instead, the signalling pathways established during HCC cells growth are more complex, and our study shows that PLAGL1 plays an important role.

PLAGL1 also induces the transcription of the *PPARy* gene [47], and by its protein could inhibit cellular proliferation through p21 regulation [17, 48]. Therefore, we studied the transcriptional activity of the *PPARy gene* during cell proliferation. We found that after releasing cells from serum starvation, its mRNA level decreased respect to that found at T = 0 hs (Figure 5). Interestingly, only SkHep1 cells exhibited genomic losses at the region where *PPARy* maps, and consequently had the lowest level of *PPARy* transcripts respect to the other tumor cell-lines (Table 1 and Figure 4C). This notwithstanding, the reduction of the transcript level is most likely related to its function as suppressor of cell division and survival [49–51]. However, some gene activity seems to be needed to maintain cell-adherence during proliferation, since it was demonstrated in HCC cells that treatment with *PPARy* inhibitors or gene silencing with small interfering RNAs cause cell detachment and anoikis [52, 53]. In this context, the low, but steady levels of PLAGL1 protein during HCC cells proliferation could also be associated with maintaining this necessary *PPARy* gene activity.

## MATERIALS AND METHODS

### Cell lines culture and tissue samples

The human hepatoma cell lines HepG2, Huh7, PLC/PRF/5 and SkHep1 derived from patients with HCC were acquired from American Type Culture Collection. Considering that the PLAGL1 expression level in fibroblasts and hepatocytes, are similar (http://genecards.org), normal fibroblasts at low passages established in our laboratory, were used as control. Cells were cultured in Dulbecco’s Modified Eagle’s Medium/Nutrient Mixture F-12 Ham (DMEM) (Sigma-Aldrich) containing 1% penicillin/streptomycin and 10% fetal bovine serum (FBS) (Invitrogen) at 37° C, constant humidity and 5% CO_2_. All experiments were performed with cells at low passages.

Seven samples from patients with HCC, one with Cholangiocellular Carcinoma (CCC), two with Hepatoadenocarcinoma (HAC) and one with Focal Nodular Hyperplasia (FNH) were collected at the Italian Hospital of Buenos Aires in accordance with the ethical standards approved by the institutional Ethical Committee and the informed written consent of the patients. Liver tissue from patients without cancer was used as control. The samples were preserved at −70° C until their use.

### Quantitative genomic aberrations by Array-CGH analysis

DNA extraction was performed using a commercial kit, according to the manufacturer’s instructions (Qiagen). DNA from healthy individuals (Promega), was used as reference for the hybridization assays. Array-CGH (aCGH) analyses were performed as previously described by Royo *et al.* [54].

### Methyl specific PCR (MS-PCR)

Genomic DNA from each tumor cell line (500ng), was subjected to bisulfite treatment, which converts unmethylated cytosines to uracils and leaves methylated cytosines unchanged, using the EZ DNA Mehtylation-Direct kit (Zymo). Specific sets of primers for MS-PCR, designed to amplify a region of the P1 promoter containing a methylated or unmethylated CpG island, were published by Leal *et al.* [55]. The PCR reaction contained 200 µmol/L of dNTPs, 200 µmol/L of MgCl_2_, 100 ng of DNA, 200 pmol/L of primers and 0.5 µl of GoTaq in a final volume of 25 µl. While the PCR conditions were as follow: initial denaturing at 94° C for 3 min, 35 cycles at 94° C for 30 sec, 51° C for 45 sec and 72° C for 30 sec, and a final extension for 5 min at 72° C. The PCR products were separated in a 10% polyacrylamide gel, stained with SYBR Green (0.0001% solution in loading buffer), and images were acquired in a gel documentation system equipped with a CCD camera.

### Proliferation assays and flow cytometry

Each cell line was plated onto 75 cm^2^ flasks in DMEM medium containing 10% FBS, and cultured until reaching 80% confluence, and then the cells were incubated in DMEM medium containing 3% FBS for 24 h. After this period of cell synchronization, four flasks for each cell line were seeded with aliquots of 25 × 10^3^ cells/ml and cultured in DMEM medium containing 10% FBS for 24, 48, 72 and 96 h, respectively. At each time point, the cells were harvested, aliquots were stained with trypan blue, and the number of viable cells determined in a Neubauer chamber. The doubling time (DT) and the constant rate of each cell line was determined using the exponential growth function (Y = Y_0_^*^exp(k^*^X)).

For cytometry analysis, an aliquot of cells from each time point of the proliferation curve was washed in PBS 1X and fixed in 1ml of ethanol 70% (ice-cold) for 30 min. Then, the cells were washed twice by centrifugation at 4000 rpm for 10 min at 4° C in PBS 1X. After centrifugation, the pellet was directly treated with a RNAse solution (100 µg/ml), and stained by adding 400 µl of propidium iodide (2 µg/ml) per million cells during 15 min. The samples were then examined in a BD FACS Canto flow cytometer and the data analysis was performed with the FlowJo 10 computer program. All samples were analysed in duplicate of two biological replicates.

### Quantitative RT-PCR

Aliquots of the cells collected at each time point of the proliferation curve were used for RNA extraction by the TRIzol method (Invitrogen), according to the protocol provided by the manufacturer. RNA concentration was measured in a Nanophotometer TM P-Class (IMPLEN). Reverse transcription of 1µg of RNA to cDNA was performed using a commercial kit (Invitrogen) in a thermal cycler (Eppendorf). The conditions were as follow: incubation at 65° C for 5 min, followed by incubation at 37° C for 52 min and at 72° C for 15 min.

To study the transcript level of the *PLAGL1, p53* and *PPARy* genes, aliquots of 70 ng of cDNA, 10µl of the MixSso (BIO-RAD), 1µl of the Probe + Primer 20X and 2 µl of H_2_O were used for each reaction in an Opticon2 instrument. The optimum quantity of cDNA (70 ng) for the detection of the transcripts and the efficiency of the technique was determined by the performance of the standard curve. To determine the efficiency of the technique, the slope of the line was calculated from the calibration assay of the standard curve of each transcript. This efficiency (≈90%) was obtained through the application of the formula: E = 10^(-1/slope)^-1. The conditions of the PCR were as follow: incubation at 96° C for 2 min; followed by 50 cycles at 96° C for 15 sec, 60° C for 1 min and 72° C for 1 min. Primers of *PLAGL1* (Assay N° H00414677_m1), *p53* (Assay N° H01034249_m1), *PPARy* (Assay N°H01115513_m1) and *PPIA*, the reference gene, (Assay Reagents Human PPIA P.N. 4333763F), were synthesized by Life Technologies. All samples were analysed in triplicate from two biological replicates. The analysis of the data was performed using the DNA Engine Opticon2 software (Gene Expression Analysis for iCycleriQ Real Time PCR detection System – BIO-RAD). For each gene, in each sample, a value of threshold cycle (Ct) was obtained, established as the number of cycles in which the reaction reaches the value of 0.02 of fluorescence intensity. This value of Ct corresponds to the average of the three values obtained in the technical replicates. The transcript level of *PLAGL1*, *p53*, *PPARy* and *PPIA* was quantified using the 2^-ΔΔCt^ method (ΔΔCt = [Ct_Target gene_– Ct_*PPIA*_]_experiment_ − [Ct_Target gene_– Ct_*PPIA*_]_control_).

### Western blot

Aliquots of cells from each time point of the proliferation curve were washed by centrifugation in PBS 1X, treated with lysis buffer containing 50 mM of Heppes, 150 mM of NaCl, 0.5 mM of EDTA, 1% of Nonidet P-40 and 10 µl of protease inhibitors/1 ml of buffer (100X, Thermo Scientific) for 30 min and centrifuged at 12000 rpm for 30 min at 4° C. The concentration of proteins in the total lysate was determined by the Bradford protein assay. The Bradford reagent consists of 1 volume of 0.02% Coomassie Brilliant Blue (G-250) dissolved in ethanol, 2 volumes of H3PO4 and 17 volumes of distilled water. The calibration curve for this assay was prepared with serial dilutions of bovine serum albumin. The solution for each measurement contained 10 µl of total lysate, 10 µl of distilled water and 200 µl of Bradford reagent. Once the protein concentration was determined, aliquots of 10 µg of total lysate were mixed with loading buffer, heated at 95° C for 5 min, and then loaded onto 10% SDS-polyacrylamide gel and separated at 100 V during 90 min. Then, the proteins were transferred onto 0.45 µm nitrocellulose membrane, blocked in 5% milk overnight at 4° C and then incubated 2 hours with primary antibody anti-PLAGL1 (1:500, sc-22811, Santa Cruz Biotechnology), anti-p53 (1:500, sc-1311, Santa Cruz Biotechnology) and anti-p21 (1:500, sc-397, Santa Cruz Biotechnology). After hybridization with primary antibody, the membrane was washed with Tris-buffered saline containing Tween-20 three times, then incubated with horseradish peroxidase-labeled secondary antibody (Santa Cruz Biotechnology) for 1 hour at room temperature and washed with Tris-buffered saline containing Tween-20 three times. Final detection was performed with ECL Chemiluminescent Western blotting reagents (Thermo Fisher Scientific). An antibody against Actin (1:500, sc-1615, Santa Cruz Biotechnology) was used for gel-loading control. Western blot signals were quantified using ImageJ program. The intensity of each band was normalized respect to that of actin (loading control) at the same time point. The assay was performed from two biological replicates.

### PLAGL1 expression *in situ*

To study the expression level of PLAGL1 and p21 proteins *in situ*, we performed immunofluorescence, immunocytochemistry and immunohystochemistry. The cell-lines were grown directly onto glass coverslips under the same conditions used for the other assays and harvested simultaneously with the cells used for qRT-PCR and Western blot. The coverslips were washed in PBS 1X, and the cells fixed in paraformaldehyde (2% in PBS 1X) at 4° C for 15 min. Endogenous peroxidase activity was quenched by the incubation of the slides in 4% hydrogen peroxide solution in PBS 1X for 40 min. The slides were washed in PBS 1X and incubated for 1 h in 3% bovine serum albumin to block unspecific sites, and then incubated with a 1:100 dilution of anti-human PLAGL1 and anti-human p21 overnight at 4° C. The detection was performed with a 1:200 dilution of secondary antibody conjugated to HRP for 1 h at room temperature. Then, slides were washed in PBS 1X and developed in 3,3′-diaminobenzidine (Liquid DAB + Substrate Chromogen System, Cell Marque). Cells were counterstained in Mayer’s hematoxylin, washed in PBS 1X, dehydrated in an ethanol series, cleared in xylene, and mounted with a solvent-based mounting medium (ALUN Metraquímica). All cell lines were stained in duplicates, and negative controls were prepared by excluding the primary antibodies in the procedure.

For immunofluorescence, the coveslips were treated similarly but excluding the incubation in hydrogen peroxide, using secondary antibodies conjugated to Alexa Fluor 546 (red) and Alexa Fluor 488 (green) and mounted with Vectashield-DAPI^®^.

PLAGL1 immunoreactivity was also analysed in 11 tissue-samples from patients with hepatic tumors, as well as non-tumoral liver tissue. Immunohistochemistry was performed on 4 µm thick paraffin sections that were deparaffinized and rehydrated in an ethanol series. Antigen retrieval was carried out by microwaving for 15 min in citrate buffer, pH 6.0 (10 mM citric acid, 10 mM sodium citrate), cooled and washed twice in PBS 1X for 5 min. The histological preparations were permeabilized with 0.05% Triton in PBS 1X, washed once in PBS 1X and next incubated in 4% hydrogen peroxide solution in PBS 1X for 40 min. Then, the same antibodies treatment and detection procedure were used as described before. The immunostained sections were evaluated using a Olympus light microscope. Protein immunostaining intensity was scored as: strong (+++), moderate (++), weak (+) and negative (−).

### PLAGL1 protein overexpression

To study the effect of PLAGL1 overexpression on HCC cells proliferation, we performed transfection assays in the PLC/PRF/5 and HepG2 cell lines with the plasmid prk7-PLAGL1 (kindly provided by Dr Laurent Journot, Institut de Genomique Fonctionnelle, Universite de Montpellier, France) [56]. These cell lines were chosen because HepG2 is normal as regard to *p53* gene, while PLC/PRF/5 presents a mutation in this gene (R249S) that encodes for a non-functional p53 protein. Aliquots of 5 × 10^4^ cells were cultured onto 6-well plates in complete DMEM medium for 72 h. At this time point (T = 0 hs; 70% confluence), the cells were transfected with the plasmid prk7-PLAGL1, using Lipofectamine 2000 (Invitrogen) following the manufacturer’s protocol. After a transfection period (6 hs) without sera, the medium was replaced by DMEM containing 10% FBS and incubated for 30 aditional hs. Controls included non-transfected cells harvested at T = 0 hs and T = 30 hs, as well as cells transfected with the empty vector plasmid.

The cells from each experimental condition were harvested, aliquots were stained with trypan blue, and the number of viable cells determined in a Neubauer chamber. PLAGL1 and p21 proteins levels were determined by Western blot and inmunfluorescence following the protocol described above. The experiments were carried out in duplicate.

### Statistics

All statistical analysis and graph were performed with Graph Pad Prism v.5.0. Comparisons between samples were evaluated with analysis of variance (ANOVA). Diferences between results were considered significant if the *p*-value was < 0.05 (^*^); < 0.001 (^**^) and < 0.0001 (^***^).
